# Peer Mentoring at the Uganda Cancer Institute: A Novel Model for Career Development of Clinician-Scientists in Resource-Limited Settings

**DOI:** 10.1200/JGO.17.00134

**Published:** 2018-03-23

**Authors:** Warren Phipps, Rachel Kansiime, Philip Stevenson, Jackson Orem, Corey Casper, Rhoda A. Morrow

**Affiliations:** **Warren Phipps**, **Corey Casper**, and **Rhoda A. Morrow**, University of Washington; **Warren Phipps**, **Philip Stevenson**, **Jackson Orem**, **Corey Casper**, and **Rhoda A. Morrow**, Fred Hutchinson Cancer Research Center; **Corey Casper**, Infectious Diseases Research Institute, Seattle, WA; and **Rachel Kansiime** and **Jackson Orem**, Uganda Cancer Institute, Kampala, Uganda.

## Abstract

Cancer centers are beginning to emerge in low- and middle-income countries despite having relatively few oncologists and specialists in related fields. Uganda, like many countries in sub-Saharan Africa, has a cadre of highly motivated clinician-scientists-in-training who are committed to developing the capacity for cancer care and research. However, potential local mentors for these trainees are burdened with uniquely high demands on their time for clinical care, teaching, institutional development, advocacy, and research. Facilitated peer mentoring helps to fill skills and confidence gaps and teaches mentoring skills so that trainees can learn to support one another and regularly access a more senior facilitator/role model. With an added consultant component, programs can engage limited senior faculty time to address specific training needs and to introduce junior investigators to advisors and even potential dyadic mentors. Two years after its inception, our facilitated peer mentoring career development program at the Uganda Cancer Institute in Kampala is successfully developing a new generation of researchers who, in turn, are now providing role models and mentors from within their group. This program provides a practical model for building the next generation of clinical scientists in developing countries.

## INTRODUCTION

The Uganda Cancer Institute (UCI)/Hutchinson Center Cancer Alliance (Alliance) was established in 2008 between the UCI and the Fred Hutchinson Cancer Research Center in Seattle, WA, to build capacity for collaborative clinical research on the pathogenesis, epidemiology, detection, and treatment of cancers, especially those related to infectious agents.^1,2^ The early years of the Alliance focused on building infrastructure for clinical research, including the provision of a variety of training experiences for promising Ugandan clinician-scientists. Eleven trainees completed a 13-month fellowship in Seattle that included didactic training in epidemiology, statistics, and the responsible conduct of research along with clinical rotations at the Seattle Cancer Care Alliance. These trainees have subsequently developed research projects toward a degree in programs at Makerere University or the University of Washington.

Continued career development of trainees in academic medicine traditionally is aided by dyadic mentoring—the ongoing collaborative interaction between the trainee and a more-experienced committed clinical scientist.^3,4^ However, geopolitical and cultural changes late in the past century left Uganda with a relatively small population of mid- and late-career medical scientists available for this type of mentoring commitment.^5^ According to in-country surveys, senior scientists and faculty members have insufficient time or training to meet the mentoring needs of the growing number of students and postgraduate trainees who attend programs of Makerere University’s College of Health Sciences, one of the premier medical schools in East Africa.^6,7^ Past and present deans and several faculty members of the four schools within the College of Health Sciences (Medicine, Biomedical Health Sciences, Allied Health Sciences, and Public Health) have served in advisory roles for the funded programs that benefit the Alliance’s Ugandan trainees. However, in Kampala, the Alliance itself has only one senior faculty member whose responsibilities include the directorship of the UCI (J.O.) and one junior faculty member from Seattle who lives in Uganda (W.P.). Research mentoring of Alliance trainees by Seattle-based senior scientists affiliated with the Alliance has been limited by 10- to 11-hour time zone differences, the intermittent availability and quality of communication technology, and infrequent formal and informal face-to-face encounters.

**Fig 1:**
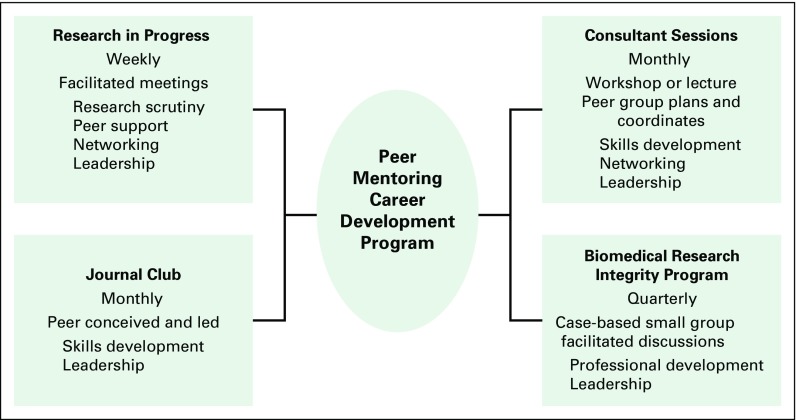
Components and goals of the peer mentoring career development program.

Facilitated peer mentoring has been described as a way to augment or replace classic mentor-mentee pairs or dyadic mentoring in academic health settings.^8-12^ In 2011, Herbert et al^13^ described an especially appealing consultancy model in which peer mentees from an interdisciplinary program in geriatric medical research select and engage senior consultants to provide in-depth and guided responses to challenges posed by the group. This approach leverages the time of senior faculty and provides leadership opportunities for peer mentees.^13^ To support the mentorship and career development of Alliance trainees and their colleagues in Kampala, we created a peer mentoring consultant program on the basis of these models. We describe the peer-driven evolution of the core consultant program into a multifactorial, peer mentoring career development (PMCD) program (Fig 1). We also offer practical suggestions for replicating such a program.

## RESEARCH IN PROGRESS MEETINGS: THE PROGRAM HUB

In response to the needs of Alliance scientists-in-training, a weekly research in progress (RIP) meeting was started in 2010. Regular attendees (peer mentees) include Alliance trainees and research personnel as well as UCI medical officers, nursing officers, and pharmacists.^14^ RIP meetings provide a safe, nonjudgmental environment for peer mentees to discuss scientific and logistical questions that arise in research projects and to practice the presentation of their research ideas and findings. The meetings also provide a forum for trainees to interact with visiting scientists. Initially, RIP sessions were facilitated by one of the authors (W.P.), but by 2013, as many as 20% of weekly sessions were facilitated by one of several Alliance peer mentees. To maintain a safe and relatively intimate environment, the RIP meetings generally were not broadcast remotely, but trainees or faculty occasionally joined sessions through Skype (Skype Communications, Luxembourg City, Luxembourg) or WebEx (Cisco WebEx, Milpitas, CA) if requested.

In 2013, a confidential survey was administered to 38 individuals who were regular attendees of RIP meetings. Of the 38 individuals surveyed, 24 (63%) stated that they were currently involved in research, 25 (66%) had attended most of the RIP sessions to date, and 36 (100% of 36 responders) agreed that RIP meetings are a good use of their time. Furthermore, 35 of 36 (97%) who responded agreed that they would like to have more mentoring and career development topics included in the RIP meetings.

The survey also revealed that only half of responders (19 of 38 [50%]) believed they had opportunities to develop leadership skills at work. Only eight of 38 (22%) expressed they were satisfied with their progress in finding a mentor, and just three of 38 (7%) believed that they had many opportunities for networking with other researchers.

## PEER MENTORING CONSULTANT PROGRAM

To address the apparent gaps in mentoring, leadership development, networking, and skills mastery, we (W.P. and R.A.M.) conducted a 2-hour workshop in Kampala in 2013 with 17 peer mentees to identify group values, agree to basic goals, and create a list of career development topics or concerns that the mentees hoped could be addressed by senior consultants. We brainstormed in small groups of two or three attendees and compiled the group suggestions to assure that all peer mentees were represented. The groups unanimously reported teamwork as a top value and set goals that included continuing weekly RIP meetings, starting a consultant program for skills building, and using the consultant program to increase networking opportunities (Table 1).

**Table 1:**
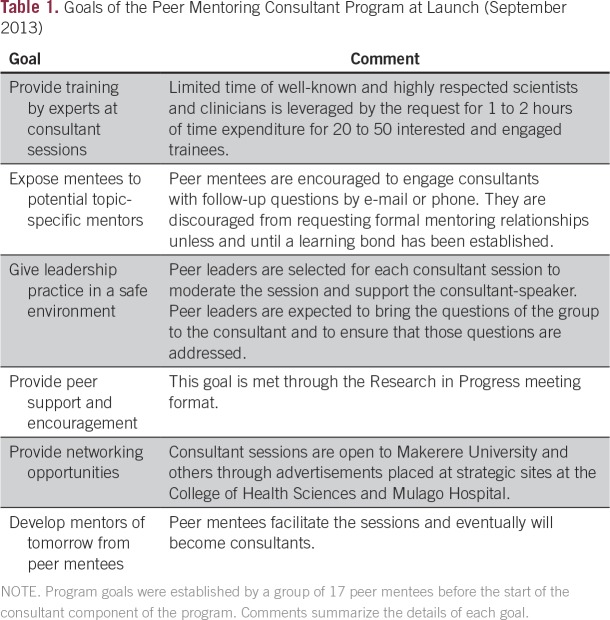
Goals of the Peer Mentoring Consultant Program at Launch (September 2013)

Workshop attendees identified 31 topics for possible future consultant presentations; topics included research design and implementation, career management, and professional skills training (Table 2). One of the authors (R.A.M.) then interviewed the deans of the four schools within the College of Health Sciences at Makerere University, who provided the names of 58 senior faculty or scientists to serve as consultants for one or more of the topics identified by the founding peer mentee group.

**Table 2:**
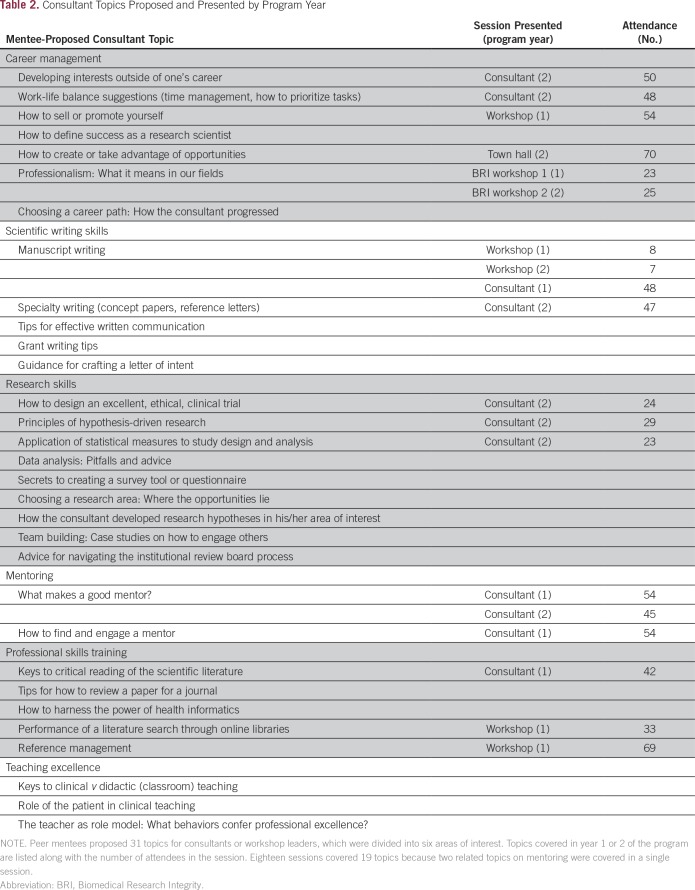
Consultant Topics Proposed and Presented by Program Year

In the first 2 years of the program, the peer mentee group held nine didactic consultant sessions, eight skills workshops, and one town hall meeting. Of these 18 sessions, nine (50%) involved local Ugandan consultants, and the remainder were led by faculty from Seattle. In general, a peer mentee volunteers to contact the consultant before the session to discuss the questions the group hopes to have addressed. The peer mentee also facilitates the session. Consultants often use slides for their talks but are encouraged to interact with the peer mentees and to elicit discussion. Slides or supplementary materials are posted, with permission, to an open-access Web page maintained by the Fred Hutchinson Cancer Research Center’s Arnold Library in Seattle.^15^

Attendance for the 15 open sessions ranged from 23 to 70 (median, 45), whereas attendance for three closed sessions ranged from seven for each of two manuscript writing workshops to 23 for a workshop on how to interpret statistics in scientific manuscripts. With permission of the consultant, evaluations are elicited at the end of each session (Data Supplement). Nearly all respondents (92% to 100%; median, 96.7%) ranked the sessions as very useful—the highest rating. Responses to the question, “What will you do differently as a result of having attended this session?” often revealed that attendees planned to incorporate new skills or practices described by the consultant. Overall, satisfaction with the sessions was high, with comments such as “make them more frequent” and “it’s fantastic” in response to the question about how the consultant sessions could work better.

## JOURNAL CLUB

Within the first year of the program, peer mentees had received a substantial amount of training in literature search and scientific reading skills. One of the peer mentees, an Alliance medical officer with ongoing research interests, independently started the Journal Club by using one of the regular RIP sessions for the time and venue. The program provided questions that were based, in part, on course materials from the first consultant session (How to Read the Scientific Literature). The peer mentee uses these questions to guide the critical evaluation of the study design, the quality of the statistical approach and data presentation, and the validity of the conclusions stated in the selected article. Since mid-2014, when Journal Club began, the group has maintained a monthly schedule, with peer mentees selecting an article of interest and a discussant for each session.

Evaluations are not elicited after Journal Club sessions; however, we did ask about the Journal Club in surveys administered to the overall peer mentoring group at the end of 2015, when the club had been active for 18 months. Most respondents (45 of 57 [79%]) indicated that they had attended Journal Club, with more than half (25 of 43 [58%]) reporting that they had attended three or more monthly meetings. The majority of Journal Club attendees (36 of 44 [82%]) believed that the club helped them to read the scientific literature more skillfully; 82% (36 of 44) believed that the club affects their clinical practice or research thinking, and 89% (40 of 45) agreed that the club was a good use of time.

## BIOMEDICAL RESEARCH INTEGRITY WORKSHOPS

Midway through year 1, in response to peer mentee requests and with additional funding, we launched a workshop series to increase understanding of ethical research and clinical practice. Biomedical research integrity (BRI) workshops take place once a quarter and use a case-based, small group format in which attendees practice bioethical decision making. The facilitator leads a group discussion on the basis of prepared concepts or guidelines for each case. In the first 2 years of the PMCD program, the BRI sessions covered six topics, including human participants and medical ethics, ethical authorship, ethical mentorship, laboratory practice, and data integrity. In contrast to Journal Club, Seattle-based Alliance faculty members write the cases for presentation and facilitate these workshops. An evaluation form is distributed after each session to probe the acceptability of the format, usefulness of the topic, and to elicit desired topics or changes to the BRI sessions. Of 58 peer mentees surveyed in 2015, 33 (57%) had attended one or both of the two most recent BRI sessions. Of these attendees, 32 (97%) believed that these workshops were a good use of their time.

## PROGRAM OUTCOMES

As the PMCD program evolved, we attempted to measure its effectiveness through surveys after each consultant or BRI session and by comparing responses to scaled surveys conducted at the beginning of the consultant program (baseline) in 2013 and at 1 and 2 years later. Thirty-eight members of the peer mentee group responded to the baseline survey; 43 responded to the 1-year survey, and 58 responded to the 2-year survey in 2015. The increased number of respondents over time reflects growing involvement in the program, which could be partly due to the increase in the number of UCI medical personnel and Alliance trainees, the opening of a new Alliance building with a larger training venue, and/or increased awareness of the PMCD program. The confidential surveys contained several questions (with yes, no, and not applicable answers) to determine attendance history and satisfaction levels with components of the program. Questions with scaled answers were included to measure confidence, leadership, mentoring, and career development. Answers to each survey were entered into SurveyMonkey (SurveyMonkey, San Mateo, CA). Student’s *t* test and χ^2^ test were used to analyze selected outcomes measures. Results of the 2014 survey have been reported.^16^ Here, we report the results of the 2015 survey taken 2 years after beginning the consultant component of the PMCD program. Journal Club had been in place for 18 months, and the BRI workshops had been offered for 10 months.

In the 2013 baseline survey, 63% (24 of 38) indicated they were involved in research, as did 78% (32 of 41) in 2014 and 65% (37 of 57) in 2015. The post-2-year survey in 2015 revealed that 18 of 55 (33%) respondents had attended RIP meetings for 1 to 3 months, 14 (25%) for 4 to 6 months; eight (15%) for 6 to 12 months, and 15 (27%) for > 12 months. As we found in the baseline survey, RIP meetings were considered a good use of time in the post-2-year survey (51 of 55 [93%]), with four of 55 (7%; all new attendees of < 3 months) stating they were unsure.

Of the 19 scaled questions in the 2013 survey, a number were repeated in subsequent surveys. Table 3 lists the weighted averages of responses to these questions among three groups: the baseline survey responders (n = 38), new attendees who joined the program within 3 months of the 2015 survey (n = 18), and veterans who indicated having been in the program for > 12 months before the 2015 survey (n = 15). In general, new attendees had similar scores as respondents to the baseline survey. Measures of a sense of community and confidence were slightly higher in the baseline group than in the new-attendees group in the 2015 survey. This finding could be explained by the fact that a majority (25 of 38 [63%]) of the baseline respondents had attended most of the RIP sessions in the previous year before the formal program launch and were highly satisfied with those meetings.

**Table 3:**
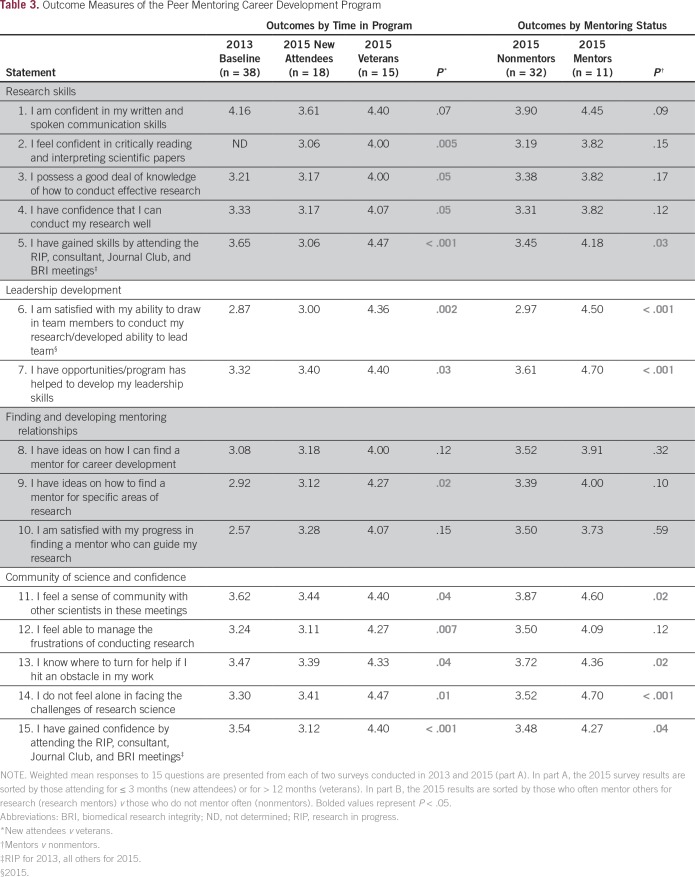
Outcome Measures of the Peer Mentoring Career Development Program

In 2015, veterans (> 12 months in program) scored significantly higher than new attendees, especially in areas covered specifically by didactic or workshop sessions, such as research skills and mentoring. For instance, veterans agreed more strongly than new attendees with the statement “I feel confident in critically reading and interpreting scientific papers” (mean, 4.00 *v* 3.06; *P* = .005), perhaps reflecting skills gained through Journal Club as well as consultant sessions in the professional skills training and scientific writing skills categories (Table 2).

Overall, the PMCD program seems to have influenced a sense of leadership ability in veterans. Veterans reported higher agreement than new attendees with the statement “The program has helped develop my leadership skills” (mean, 4.40 *v* 3.40; *P* = .03) and reflected much higher agreement than new attendees with the statement “I have developed the ability to lead a team” (mean, 4.36 *v* 3.00; *P* = .002).

The measures of sense of community and confidence in science, although relatively high at baseline and in new attendees, were highest in veterans of the program. For example, veterans more strongly agreed than new attendees with the statements “I do not feel alone in facing the challenges of research science” (mean, 4.47 *v* 3.41; *P* = .01) and “I feel able to manage the frustrations of conducting research” (mean, 4.27 *v* 3.11; *P* = .007).

## MENTEES BECOME MENTORS

The ultimate goal of a PMCD program like ours is to graduate peers to roles of mentorship within their own independent research or clinical endeavors. In the 2015 survey, we sought to determine a baseline of mentoring activity within the group so that we could determine in later surveys whether mentees were indeed converting to greater mentoring roles. A substantial proportion of respondents reported that they often mentored others for clinical issues or practice (29 of 43 [62%]), for career development (12 of 43 [27%]), or for research-related issues (11 of 43 [26%]). Because our Alliance emphasizes growth and independence in research, we were particularly interested in learning more about those who were often research mentors. Not surprisingly, those who often mentored for research issues were more likely than nonmentors in research to have been in the program for > 12 months (nine of 11 [82%] *v* five of 32 [17%]). Research mentors were more likely to have attended most (> 50%) of the RIP sessions in the previous year (six of 11 [55%] *v* 11 of 32 [39%]) and to have attended Journal Club more than four times in the previous year (six of 10 [60%] *v* 12 of 23 [38%] of those attending).

We compared weighted responses from the 11 research mentors with the 32 who sometimes (17 of 43 [39%]), rarely (seven or 43 [16%]), or never (eight of 43 [19%]) mentored for research-related issues (nonmentors). These two groups had similar scores for confidence in their communication skills, research ability, and research knowledge. The groups had similar scores for topics related to having ideas for finding mentors and for their level of satisfaction in finding their own mentors (Table 3). However, research mentors had significantly higher scores in their confidence for reading and interpreting scientific articles and in having gained skills through attending PMCD program meetings (mean, 4.18 *v* 3.45; *P* = .03). They also had a greater sense of community by several measures and were more strongly in agreement that they had gained confidence by attending PMCD meetings (mean, 4.27 *v* 3.48; *P* = .04). The most striking difference was in leadership development. Research mentors believed more strongly than nonmentors that they had developed the ability to lead a team (mean, 4.50 *v* 2.97; *P* < .001) and responded with higher scores to the statement that the program had helped to develop their leadership skills (mean, 4.70 *v* 3.61; *P* < .001). In summary, those individuals who were more experienced and active in the PMCD program were more likely to start mentoring others.

## EFFECT ON RESEARCH OUTPUT

The assessment of the direct effect of our program on research output at this early stage is challenging. Still, participants in the PMCD program have many important accomplishments to suggest that the program is supporting the successful development of junior investigators. In the first 2 years of the program, peer mentees participated as study nurses, study doctors, or as investigators in > 25 research studies conducted at UCI. Trainees presented 15 abstracts at international conferences on the basis of their work during this time. Several PMCD participants also enrolled in higher degree programs, including two who completed master in public health degrees and five who are currently pursuing doctorate in philosophy degrees. Five peer mentees successfully obtained independent grant funding in the first 2 years of the program, including two Beginning Investigator Grant for Catalytic Research awards from the National Cancer Institute. Furthermore, seven peer-reviewed articles were published by program participants by the second year of the program, and several more have been published since. 

In addition to focusing on research skills development, the PMCD program emphasizes leadership development. In the 2 years after the period of this study, peer mentees have assumed several leadership positions within the UCI, including director of clinical services, director of community outreach, director of satellite clinical services, founding secretary of the UCI institutional review board, and head of the UCI research and training directorate. One peer mentee has also assumed the directorship of the clinical microbiology laboratory at Makerere University.

## CREATION OF A PMCD PROGRAM

For those who are considering starting a peer mentoring program, we would advise that the following elements be incorporated. First is a core identity or underlying common purpose as identified by the group itself. For new cancer centers in low- and middle-income countries, peer groups might easily arise from the common challenges faced by clinicians new to oncology or by a group of early-stage scientists who are undertaking cancer-related research projects. Participation in the program should be voluntary to ensure that group members have an interest in research and skills development. 

The second element is staff time for logistics and communication. Having ongoing assistance to track attendance, register attendees for consultant sessions, send meeting reminders, and administer evaluations is invaluable. Small groups may be able to rotate these duties, but even routine functions can become burdensome to busy trainees or junior faculty.^9^ Protected support staff time, which required approximately 0.25 to 0.5 full-time equivalents for our program, is helpful for supporting program communications, tracking, and evaluation. 

The third element is having a senior facilitator or advisor—a key element for new groups to help to establish goals, to be a role model and advocate, and to provide counsel to keep the group going in the early stages.^17^ Ideally, this advisor would reside in the country to attend program activities and provide regular face-to-face interactions with the group. 

The fourth element is to augment, but not manage, existing or nascent mentoring dyads. Some trainees had existing mentoring relationships, particularly those enrolled in formal degree programs, whereas others did not. Our PMCD program was not designed to assign mentors to trainees but, instead, to provide guidance on how to identify a mentor and how to manage the mentor-mentee relationship. Each individual peer mentee was responsible for managing exchanges with mentors outside the group. 

The fifth element is to empower peer mentees. Trainees in countries with a relatively collective rather than individualistic viewpoint of group interaction^18^ may thrive within relaxed programmatic parameters that depend more on relationship and trust building than on rules. Our PMCD program allows individuals to participate as much or as little as their schedules and interests allow. Most peers express pride in the PMCD program and engage in regular reviews to revise goals, identify needs, and brainstorm changes they want to implement. 

The final element is to encourage organic transformation. Peer mentee empowerment leads, naturally, to programmatic changes. Alliance faculty members support such changes, a factor that may encourage peer mentee engagement.

## DISCUSSION

The PMCD program emanated from a weekly facilitated meeting of peers who had a common interest in cancer care and cancer-related research. The program has evolved quickly, but organically, to encompass four separate but interrelated training and leadership modalities (Fig 1). The program was initially conceived to respond to the limited senior faculty available^6,7^ to mentor junior scientists with their research projects within the Alliance.

Peer mentoring within groups of junior faculty arose in response to the lack of senior faculty availability for classic dyadic mentoring.^9,10^ The conceptual framework and motivation of our PMCD program draws heavily from the rationale by Pololi et al,^8,17^ who emphasized the importance of applying adult learning precepts to what they termed collaborative mentoring. In collaborative mentoring, a supportive learning environment is coupled with learner engagement. This approach follows Rogerian principles of adult learning in which a safe, nonjudgmental learning environment is stressed and learning occurs through experiential opportunities.^8,19^ The RIP meetings create a supportive setting, whereas the consultant program emphasizes group empowerment by having the peer mentees identify their group’s values, develop their own learning and program goals, and take responsibility for implementing the program.

Peer mentoring has the advantage of reducing the power differential inherent in dyadic mentoring relationships.^4^ Potential disadvantages include the development of professional or personal rivalries within the group as individuals strive toward recognition and promotion. To this point, our group seems distinct from those described in North American academic medical systems.^9^ A values exercise held during the planning meeting for the consultant program revealed that teamwork and kindness/charity were the two most frequently stated values. These values have been reflected in mutual peer respect and overall commitment to the group’s success. As a result, we have not encountered difficulties with intragroup competition as individuals develop in their careers.^4^ A second potential limitation of peer mentoring is the inherent lack of experience of the mentees with their related inability to provide networking to their junior scientist–level peers. The consultant system was designed specifically to expose peer mentees to a variety of vetted, senior faculty members and to provide introduction points to potential dyadic mentorship opportunities. To encourage networking, the program has had an open-door policy with widely advertised consultant sessions for all but three of the 18 didactic presentations or skills workshops in its first 2 years.

The structure, content, evaluation process, and support mechanisms of our PMCD program place responsibility for career development largely on the individual trainee. This concept, derived from business precepts^20,21^ and as interpreted for careers in academic medicine,^22^ is especially well suited for resource-challenged institutions where organized, dyadic mentoring programs may not be feasible. Along with self-management of a trainee’s career track, as facilitators (W.P., R.A.M.), we emphasize the importance of seeking multiple situational mentors for focused advice. Each local expert who has served as consultant has invited attendees to follow up with them for more-specific advice. Anecdotally, these invitations have been accepted in a number of cases. Limited studies indicate that mentoring dyads that connect organically can become both long lasting and meaningful.^17^

Comparison of baseline and post-2-year survey data suggests that veteran (> 12 month) program participants show increased confidence and heightened perceived ability to find mentors. Furthermore, veterans have significantly higher scores than program newcomers for leadership measures and for a sense of community. Perhaps most tellingly, a substantial number of veteran peer mentees have assumed roles as mentors for others in research endeavors.

Our survey techniques had a number of limitations. Because they were designed to provide feedback that would be useful to build the program, we did not track who took either survey and, therefore, could not make direct assessments of how the program influenced individual participants. Furthermore, because we wished to have anonymous responses, we avoided having respondents identify their occupations or the depth of their involvement with research. Although this method allowed for frank comments (and hopefully, honest responses), biases may have been introduced by regularly attending group members whose professional needs are not the focus of the program. For example, several regular attendees are highly experienced and professionally confident senior nurses who have limited interest in specific aspects of research skills training. Our use of veteran or long-term attendees would also select for those who found the program useful enough to continue participation. Conversely, some of those who responded to either survey may have had only passing exposure to the program. Finally, we had no way to determine who may have been missed in the surveys. In particular, we did not attempt to track those who had attended and then accepted a graduate or professional position outside Kampala. Their input about the effect of the program on their careers, although potentially valuable, was not sought. The identification of dropouts and their reasons for departure would be of interest.

The initial goals of the PMCD program have been met and, in many ways, exceeded as a result of peer interest, engagement, and growing peer leadership. Peer group members are taking increasing responsibility for PMCD components as they advance in research and clinical experience, confidence, and leadership. As peer mentees advance to roles as mentors, PMCD facilitators, and consultant session leaders, we anticipate additional organic changes. Meanwhile, the basic precepts and simple organization scheme of our PMCD program may provide a roadmap for other cancer research programs to create similar effective peer mentoring groups for career development of junior faculty and research trainees.
